# Neurocysticercosis in patients with active epilepsy in the tea garden community of Assam, Northeast India

**DOI:** 10.1038/s41598-021-86823-w

**Published:** 2021-04-01

**Authors:** K. Rekha Devi, Debasish Borbora, Narayan Upadhyay, Dibyajyoti Goswami, S. K. Rajguru, Kanwar Narain

**Affiliations:** 1 ICMR-Regional Medical Research Centre, N. E. Region, Dibrugarh, Assam 786001 India; 2 Department of Biotechnology, Gauhati University, Guwahati, Assam 781014 India; 3 Department of Neurology, Assam Medical College and Hospital, Dibrugarh, Assam 786002 India

**Keywords:** Risk factors, Neurological disorders, Parasitology

## Abstract

Neurocysticercosis is a significant cause of epilepsy in the tropics. The present cross-sectional survey was conducted in the socioeconomically backward tea garden community of Assam to gauge the prevalence of neurocysticercosis in patients with active epilepsy and to determine the associated risk factors. In a door to door survey, a total of 1028 individuals from every fifth household of the study Teagarden were enrolled to identify self-reported seizure cases, followed by a neurological examination to confirm the diagnosis of active epilepsy. Patients with active epilepsy underwent clinical, epidemiological, neuroimaging (contrast-enhanced computerized tomography) and immunological evaluations to establish the diagnosis of neurocysticercosis. Clinically confirmed 53 (5.16%) active epilepsy were identified; 45 agreed to further assessment for neurocysticercosis and 19 (42.2%) cases fulfilled either definitive or probable diagnostic criteria for neurocysticercosis. Patients with epilepsy due to neurocysticercosis were more likely to suffer from taeniasis (20.0% vs 0.0%), rear pigs (57.9% vs 15.4%) or have pigs in their neighbourhood (78.9% vs 53.8%) relative to epileptic patients without neurocysticercosis. Rearing pigs (aOR 14.35, 95% CI: 3.98–51.75) or having pigs in the neighbourhood (aOR 12.34, 95% CI: 2.53–60.31) were independent risk factors of neurocysticercosis. In this community, the prevalence of taeniasis (adult worm infection) was 6.6% based on microscopy. The study reports a high prevalence of active epilepsy in the tea garden community of Assam and neurocysticercosis as its primary cause. The high prevalence of taeniasis is also a significant concern.

## Introduction

Epilepsy is a common neurological disorder that affects more than 10 million Indians^1^ and about 70 million people globally^2^. Its high incidence, especially in the developing world, can be attributed to neurocysticercosis (NCC)^3^. Neurocysticercosis is a neurological disorder that is responsible for approximately 50 thousand deaths annually and is caused by the metacestode of the parasite *Taenia solium*^4^. Humans (only definitive host), suffer from taeniasis when adult tapeworms harbour in the intestine, whereas both pigs and humans (intermediate hosts) suffer from cysticercosis when the larvae anchors in various internal organs (skeletal muscle), including the brain. Neurocysticercosis can be acquired under any socioeconomic and cultural conditions where there is close contact with a taeniasis carrier^5^. Its management becomes problematic in developing countries where sanitation, hygiene and pig management practices are poor^6^.

There are only a few studies that have evaluated the prevalence of neurocysticercosis associated epilepsy in the endemic belt (Asia, Sub-Saharan Africa, and Latin America)^3,7–12^. In 2011, the approximate annual loss of US $ 185.14 million was reported from India due to neurocysticercosis associated active epilepsy^13^. As the epidemiologic information concerning the Northeast Indian region is scanty, this study was conducted in the socioeconomically backward tea garden community of Assam to gauge the incidence of active epilepsy and estimate the burden of neurocysticercosis amongst those cases by using clinical data, serological and neuro-imaging techniques. The study also aimed to determine the prevalence of cysticercosis and taeniasis in the said community, including associated risk factors.

## Results

### Background characteristics

The mean age of the 1028 (477 male, 551 female) study subjects was 25.5 years (SD, 14.9 years). The overall socioeconomic index was low. The working class (n = 540) comprised mostly of tea garden labourers (48.5%), farmers and casual workers (29.1%). Few participants reportedly retired from work (n = 43); 421 participants (40.9%) were either studying or were unemployed. Twenty-four individuals declined from sharing their job detail. Many (17.3%; 171/988) practised traditional pig farming as a secondary occupation; 64% (658/997) consume pork and pork products in this community. The majority of families did not use a pressure cooker for boiling pork (68.5%). Hygiene was low, and the pigs were free-ranging. Open field defecation was rampant (19.8%), and many subjects used either plain water or soil to wash hands after defecation (20.0%).

### Prevalence of active epilepsy

During the door-to-door survey, 53 individuals had clinically confirmed active epilepsy; giving us a point prevalence of 5.16% (51.6/1000) with a significant difference between males (6.71%; 32/477) and females (3.81%; 21/551) (χ^2^ = 4.39; P = 0.036). The mean age of the cases was 30.6 (SD, 14.8 years). The age range of male cases was 10–64 years, and that of female cases was from 13 to 65 years. Table 1 categorizes the seizure types according to their prevalence. Partial seizure with secondary generalization was most frequent among epileptic patients, 50.9% (27/53). Headaches were the most common clinical presentation (18.9%) followed by involuntary muscle movements (7.5%) and unconsciousness (1.9%) in patients with active epilepsy.

**Table 1:** Type of seizure among patients with active epilepsy and neurocysticercosis.

Type of seizure	In all active epilepsy cases irrespective of presence or absence of neurocysticercosis (n = 53) (%)	Active epilepsy patients with neurocysticercosis (n = 19) (%)
**Generalized seizure**		
Generalized tonic clonic seizure	8 (15.1)	4 (21.1)
Complex tonic seizure	6 (11.3)	3 (15.8)
Myoclonic or atonic seizure	1 (1.9)	0 (0.0)
Absence seizure	1 (1.9)	0 (0.0)
**Partial seizure**		
Partial seizure with secondary generalization	27 (50.9)	9 (47.4)
Simple partial seizure	8 (15.1)	2 (10.5)
Complex partial seizure	2 (3.8)	1 (5.3)

### Prevalence of NCC among patients with active epilepsy

Due to funding limitations, the CT scan was available for patients with active epilepsy only. Forty-five cases of active epilepsy consented for CT evaluation, of which 19 (42.2%) had parenchymal lesions suggestive of neurocysticercosis. Ten cases presented multiple lesions, a majority of which were calcified (Table 2). One patient with multiple active lesions had Hydrocephalus. Eighteen of these 19 CT positive cases agreed for an immunological evaluation by EITB, wherein 17 (94.4%) tested positive for one or more bands < 50 kDa.

**Table 2:** CT scan features of active epilepsy patients with neurocysticercosis.

Solitary (n = 9)	Active	2 (10.5%)
	Inactive/calcified	7 (36.8%)
Multiple (n = 10)	Active	4 (21.1%)
	Degenerative	1 (5.3%)
	Inactive/calcified	5 (26.3%)
Total		19 (100%)

CT findings classified into three groups as suggested by Carpio and Escobar.

Overall, the study made 17 definitive and two probable diagnoses of neurocysticercosis among the active epilepsy cases with a male, 11/27 (40.7%); to female, 8/18 (44.4%); the ratio of 1.38:1 (p = 0.161). The mean age of the cases was 29.6 (SD, 16.0 years). The age range of the male cases was 10–64 years (median age = 32 years), and that of female cases was from 17 to 65 years (median age = 21.5 years). Epileptic patients with NCC frequently reported partial seizures with secondary generalization 9/19 (47.4%), followed by generalized tonic–clonic seizure 4/19 (21.1%) and complex tonic seizure 3/19 (15.8%) (Table 1).

### Seroprevalence of IgG antibodies against Taenia solium metacestode

Of the 1028 participants, 987 consented for an ELISA test. Two hundred seventy-three individuals (27.7%) tested positive for *Taenia solium* IgG antibodies. Among males and females, the median age at diagnosis was 30 (range 8–75) years and 27.5 (range 8–65) years, respectively. Seroprevalence was higher in male, 28.9% (132/456); than in female, 26.6% (141/531) (χ^2^ = 0.65; P = 0.40). In the community, seroprevalence increased significantly with age. Seroprevalence in the pediatric population (≤ 18 years) was 20.4% (88/432), which increased to 29.6% (99/334) in the 19–39 years age group and peaked in the ≥ 40 years age group (38.9%; 86/221) (Overall trend χ^2^ = 26.09; P = 0.000). Among ELISA positive cases, the majority presented with partial seizures with secondary generalization 16/33 (48.5%), followed by generalized tonic–clonic seizure 9/33 (27.3%) and simple partial seizure 4/33 (12.1%). Table 3 summarizes the ELISA results with and CT findings. In 26 cases, the brain CT finding was normal; however, 17 (65.4%) cases tested positive for anti-Cysticercus IgG. Among individuals with calcification as their only CT finding, multiple calcifications were more frequently associated with seropositivity.

**Table 3:** Anti-cysticercus antibodies in sera from patients with respect to number of lesions in the brain.

No. of lesions in the brain	Total cases (n = 45)	Anti-cysticercus IgG ELISA	
		No. (%) of sera tested positive	No. (%) of sera tested negative
No lesion (normal scan)	26	17 (65.4)	9 (34.6)
Solitary active	2	1 (50.0)	1 (50.0)
Solitary calcified	7	1 (14.3)	6 (85.7)
Multiple active	4	1 (25.0)	3 (75.0)
Multiple degenerative	1	0 (0.0)	1 (100.0)
Multiple calcified	5	3 (60.0)	2 (40.0)

### Diagnosis of taeniasis

The prevalence of taeniasis (adult worm infection) in the community was found to be 6.6% (33/497) with a male, 8.5% (20/236); to female, 5.0% (13/ 261) ratio of 1.53:1 (p = 0.1). Eleven cases (33.3%) suffering from taeniasis were also seropositive against *Taenia solium* cysticerci.

### Risk factors

Table 4 presents the results of the univariate logistic regression analysis to identify predictors associated with the diagnosis of neurocysticercosis among epileptic patients at study measurement. Epileptic patients with neurocysticercosis were more likely to be suffering from taeniasis (20.0% vs 0.0%), rear pigs (57.9% vs 15.4%, p = 0.005) and have pigs in their neighborhood (78.9% vs 53.8%, p = 0.089) than epileptic patients without neurocysticercosis. Table 5 presents results of the univariate and multivariate logistic regression analysis (involving 19 neurocysticercosis cases and 1009 control subjects from the study population) to identify predictors associated with the diagnosis of neurocysticercosis, together with crude and adjusted odds ratios, and 95% confidence intervals. In the cohort, those suffering from taeniasis, rearing pigs in the household and having pigs in the neighbourhood were more likely of developing neurocysticercosis. The odds of neurocysticercosis were significantly higher among those rearing pigs in their household (aOR 14.35, 95% CI: 3.98–51.75) and those having pigs in their neighbourhood (aOR 12.34, 95% CI: 2.53–60.31). Despite being statistically insignificant, our data suggest that within the cohort, males were more likely to be affected than female (OR 1.60; p = 0.315) and individuals between 20 and 39 years of age were at an elevated risk of acquiring neurocysticercosis (OR 4.69; p = 0.141).

**Table 4:** Univariate analysis showing the association of selected factors and risk of active epilepsy with neurocysticercosis vs active epilepsy without neurocysticercosis.

Category	Active epilepsy with NCC (n = 19) (%)	Active epilepsy without NCC (n = 26) (%)	Crude OR (95% CI)	P-value
**Gender**				
Female	8 (42.1)	10 (38.5)	1	
Male	11 (57.9)	16 (61.5)	0.86 (0.26–2.87)	0.805
**Age (years)**				
≤ 10	1 (5.3)	1 (3.8)	1	
11–19	3 (15.8)	6 (23.1)	0.50 (0.02–11.08)	0.661
20–39	11 (57.9)	10 (38.5)	1.10 (0.06–20.01)	0.949
≥ 40	4 (21.1)	9 (34.6)	0.44 (0.02–9.03)	0.598
**Taeniasis**^**α**^				
No	12 (80.0)	16 (100.0)	1	
Yes	3 (20.0)	0 (0.0)	9.24 (0.44–195.70)	0.153
**Rearing pigs in the household**				
No	8 (42.1)	22 (84.6)	1	
Yes	11 (57.9)	4 (15.4)	7.56 (1.86–30.71)	0.005
**Pigs in the neighbourhood**				
No	4 (21.1)	12 (46.2)	1	
Yes	15 (78.9)	14 (53.8)	3.21 (0.83–12.35)	0.089
**Consumption of pork**				
No	7 (36.8)	13 (50.0)	1	
Yes	12 (63.2)	13 (50.0)	1.71 (0.51–5.74)	0.382

Odds ratio (OR), 95% confidence interval (CI) and *P-*value were derived using univariate logistic regression analysis.

^α^Data missing in some cases which were excluded from the analysis.

**Table 5:** Predictors of neurocysticercosis in the tea garden community of Assam, Northeast India.

	Cases (n = 19) (%)	Control (n = 1009) (%)	Crude OR (95% CI)	P value	Adjusted OR (95% CI)	P value
**Gender**						
Female	8 (42.1)	543 (53.8)	Ref		Ref	
Male	11 (57.9)	466 (46.2)	1.60 (0.64–4.02)	0.315	2.01 (0.59–6.86)	0.262
**Age (years)**						
≤ 10	1 (5.3)	139 (13.8)	Ref		Ref	
11–19	3 (15.8)	320 (31.7)	1.30 (0.13–12.64)	0.819	0.38 (0.02–8.00)	0.538
20–39	11 (57.9)	326 (32.3)	4.69 (0.60–36.68)	0.141	4.19 (0.42–41.25)	0.220
≥ 40	4 (21.1)	224 (22.2)	2.48 (0.27–22.43)	0.418	3.03 (0.28–33.11)	0.364
**Taeniasis**						
No	12 (80.0)	452 (93.8)	Ref		Ref	
Yes	3 (20.0)	30 (6.2)	3.76 (1.01–14.07)	0.049	3.45 (0.60–19.726)	0.164
**Rearing pigs in the household**						
No	8 (42.1)	838 (83.1)	Ref		Ref	
Yes	11 (57.9)	171 (16.9)	6.74 (2.67–17.00)	0.000	14.35 (3.98–51.75)	0.000
**Pigs in the neighbourhood**						
No	4 (21.1)	601 (59.6)	Ref		Ref	
Yes	15 (78.9)	408 (40.4)	5.52 (1.82–16.76)	0.003	12.34 (2.53–60.31)	0.002
**Consumption of undercooked pork**						
No	7 (36.8)	341 (33.8)	Ref		Ref	
Yes	12 (63.2)	668 (66.2)	0.87 (0.34–2.24)	0.781	1.35 (0.31–5.81)	0.686

Model adjusted for all variables included in the table.

Controls were apparently healthy individuals from the community.

*OR* odds ratio, *CI* confidence interval, *Ref* reference group.

## Discussion

Epilepsy is a chronic brain disease affecting more than 50 million people worldwide. The low-and middle-income countries bear the burden of nearly 80% cases where the overall incidence rate is up to twofold higher than that of non-endemic high-income countries^2^. In India, the prevalence of active epilepsy ranges from 0.38% (3.8 per 1000 populations) in Tamil Nadu^8^ to 5.8% (58 per 1000 populations) in a pig farming community of Uttar Pradesh^9^. In the present cross-sectional study, the prevalence of active epilepsy was 5.15% (51.5 per 1000 population) which is one of the highest reported in the literature. Given the social stigma associated with epilepsy, and its variable clinical presentation, the actual prevalence of epilepsy in this community maybe even higher.

There is a large body of evidence suggesting that zoonotic and vector-borne parasites are major preventable risk factors for epilepsy in low-and-middle-income countries^14^. Exposure to or infection with *Taenia solium* larval stages including infection with *Toxocara canis, Toxoplasma gondi, Plasmodium falciparum* and HIV can manifest in the human CNS and can lead to epilepsy development^15^. Once the parasite makes its way to the brain, therapeutic interventions do not seem to influence the development of epilepsy in the long run and so strategies to control, eliminate and eradicate parasites represent the most feasible way to reduce the epilepsy burden at present^16^.

This study also demonstrates a high prevalence of neurocysticercosis among patients with active epilepsy. Forty-two per cent cases (19/45) fulfilled either definitive or probable diagnostic criteria for neurocysticercosis; which is more significant than those reported from the endemic region in Peru (35.1%)^17^, Bolivia (27.4%)^18^, Ecuador (26.3%)^19^, Tanzania (16.5%)^20^ and rest of India including Jammu (40%)^10^, Odisha (28.1%)^12^, Uttarakhand (24.8%), and Andhra Pradesh (13.7%)^7^ suggesting that NCC is a larger public health problem in this Northeast Indian community. In Zambia, however, the prevalence of neurocysticercosis among people with epileptic seizures was 57%^21^. Nevertheless, this observed incidence of symptomatic neurocysticercosis is likely an understatement as (i) 8 out of 53 (15%) individuals with active epilepsy did not agree to neurological investigations and (ii) some of the lesions may not be detected in brain CT.

Seizures are the most common presentation associated with neurocysticercosis. A predominance of generalized seizures was reported by prior studies^8,21^, but in India, seizures are often focal (partial) with or without secondary generalization^22^. In this study too, we found partial seizures with secondary generalization to be the most frequent clinical evaluation (47.4%).

In the present series, multiple lesions were more predominant (10/19; 52.6%) than single lesions (9/19; 47.3%) (Table 2). This observation was unlike most of the previous reports from India in which neurocysticercosis presents itself as solitary cysts^9,10,23,24^ but similar to reports from Latin America and China^22^. We believe that the high prevalence of taeniasis (6.6%) could be associated with autoinfection and explain for multiple lesions in the CT. However, it is interesting to note that although extra parenchymal neurocysticercosis is frequent in Latin America, CT features in our study group were parenchymatous. This variability could be related to the complex host-parasite and environmental interaction.

According to Del Brutto, calcifications are responsible for a sizable proportion of neurocysticercosis related seizures or headaches^25^. In this series, too, calcified lesions (solitary, 7/9; and multiple, 5/10) were responsible for most of the neurocysticercosis related seizures that could explain for the seronegativity observed during ELISA as calcification is associated with minimal immune response. However, 60% of the cases with multiple calcifications tested positive during anti- Cysticercus IgG ELISA.

This study examined the seroprevalence against *Taenia solium* cysticerci using both EITB and ELISA. Although EITB is a preferred choice, studies have reported that ELISA based on cystic fluid/vesicular fluid of *Taenia solium* cysticerci is highly sensitive and specific for immunodiagnosis of NCC^26^. As determined by ELISA, only 41.5% (22/46) active epilepsy patients and 31.6% (6/19) neurocysticercosis patients were seropositive.

The prevalence of *Taenia solium* taeniasis was at 6.6% (66 per 1000 populations) in the community. Three neurocysticercosis patients also tested positive for taeniasis (15.8%). There could be a possibility that human cysticercosis was associated with autoinfection and person to person transmission.

‘Open field defecation’ was rampant in the tea garden community, which could be a significant cause of faecal contamination of vegetables with *Taenia solium* eggs. Inadequate sanitation facilities also allow pigs which are in close association with humans to have easy access to human faeces contaminated with *Taenia solium* eggs. In the community, consumption of undercooked pork was also a potential risk factor. All these aspects could probably explain the high incidence of NCC in our study population.

The highest prevalence of NCC in the 20–39 years age group (11/337) with an almost equivalent male (5/165) to female distribution (6/172); probably because a majority in this age group (both sexes) were engaged in the tea plantation or practised agriculture. The seroprevalence of IgG antibodies against *Taenia solium* metacestode in the pediatric population (≤ 18 years) was 20.4% (88/432). Only four cases of pediatric neurocysticercosis were detected, and all of these cases were ELISA-negative, with solitary calcified lesions on CT. Patients with calcified lesions have significantly more seizures^27^. Singhi et al. reported that albendazole has little effect on calcification stage^28^, and children with isolated neurocysticercosis recovered without antiparasitic therapy^29^. NCC is infrequent in children^30^ probably because of age-based differences on the mode of disease acquisition and differences in immune reactivity against the parasite.

In this community, individuals may have developed immunity against *T. solium* neurocysticercosis. Table 5 outlined the fact that individuals aged 20 – 39 years were at a greater risk of acquiring NCC than individuals aged 40 years and above even though seroprevalence of IgG antibodies against *Taenia solium* metacestode was highest in the ≥ 40 years age group (86/221) than the 19–39 age group. Such age-dependent difference in the manifestation of neurocysticercosis has also been reported by others^22^. Although ELISA indicated widespread exposure to the parasite, there is a low prevalence of symptomatic human neurocysticercosis. A similar finding was reported by Jayaraman et al*.*^31^.

The limitation of this study was the small sample size. This study was a pilot study with an aim that it will form the basis of a comprehensive study. Magnetic resonance imaging (MRI) was not available, and CT could have missed small enhancing lesions oedema.

In conclusion this study reports of a strikingly high prevalence of active epilepsy in the tea garden community of Assam (51.6/1000) with neurocysticercosis as a predominant cause (42.2%). The high seroprevalence of *Taenia solium* antibodies and taeniasis (66/1000) in this community provides an insight into the predominant risk factors within the community. We believe that proper education, promotion of healthy lifestyle, behavioural motivation and safe pig husbandry practices will be essential steps that will help reduce the burden of the disease in this community.

## Methods

### Study area

**Figure 1:**
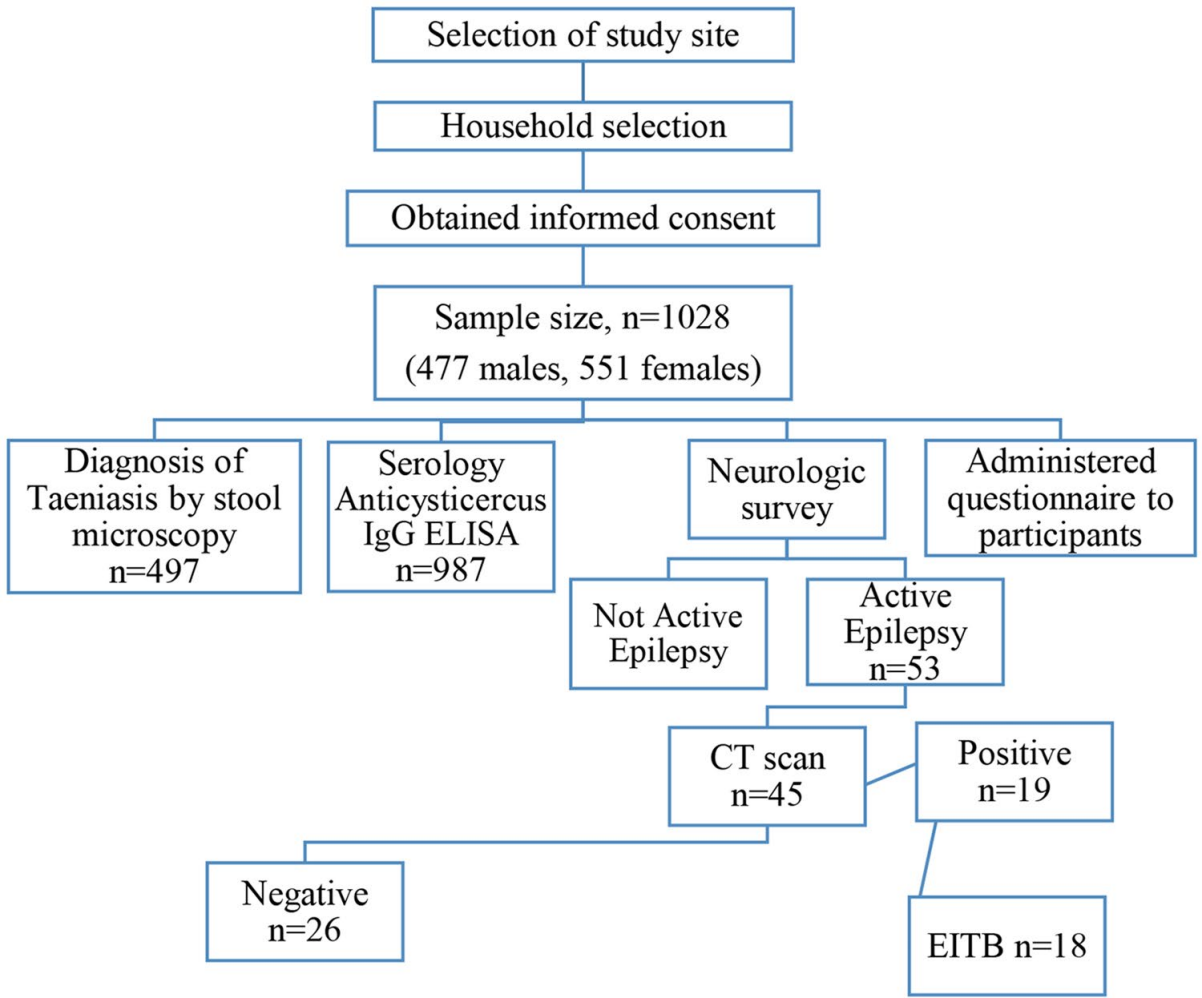
Flow diagram of the study. In a door to door survey, a total of 1028 individuals from every fifth household of the study Teagarden were enrolled to identify self-reported seizure cases, followed by a neurological examination to confirm the diagnosis of active epilepsy. Patients with active epilepsy underwent clinical, epidemiological, neuroimaging (contrast-enhanced computerized tomography) and serological evaluations to establish the diagnosis of neurocysticercosis.

A cross-sectional study was undertaken in a rural tea garden community of Basmatia tea estate, Dibrugarh district, Northeast India (Fig. 1). The study area covers 978.63 ha with more than 963 households. The population of this block is 4728 (according to Dibrugarh district census handbook, census of India 2011). Most of the members of the community belong to the low socioeconomic group and many practice traditional pig farming as a secondary occupation. Pork and pork products consumption persists in this community.

### Sample size and sampling technique

The calculation of the sample size was based on the assumption that p, the occurrence of neurocysticercosis-related seizures in this particular community would be 5% (based on our preliminary studies in the same locality). Other assumptions made during the sample size calculation were 2% marginal error (d) and a confidence interval of 95%. The calculated sample size was 456 based on the formula as follows:

$$ {\text{Sample}}{\kern 1pt} \;{\text{size}} = \frac{{Z_{1 - \alpha /2}^{2} p(1 - p)}}{{d^{2} }} $$

We took the design effect of two; thereby, the minimum sample size would be 912. Assuming 12% non-response, we planned to enrol 1028 individuals belonging to every fifth household in the community. The survey team for the door-to-door survey consisted of one clinician, two trained technicians and a health care worker to identify those with self-reported seizures and symptoms such as headaches, fainting, and fatigue, followed by a neurological examination to confirm the diagnosis of active epilepsy.

### Data collection

A precoded questionnaire was administered in the local language to collect epidemiological information including age, religion, marital status, education, family income, source of drinking water, dietary and cooking habits, personal hygiene, pig raring practices, development of subcutaneous or muscular nodules and history of passing *Taenia* proglottids. A thorough history of seizure, as well as other factors leading to epilepsy such as alcohol consumption, head trauma, diseases like tuberculosis, was also taken. Informed consent was taken from all the participants (or in the case of minors, from their guardians) in the study.

### Diagnostic criteria for active epilepsy

All individuals aged five or older who reported seizures were examined by a neurologist to confirm the diagnosis of active epilepsy. A case of epilepsy was defined as two or more afebrile seizures unrelated to the withdrawal of alcohol or drugs or to acute metabolic disorders^32^ and grouped according to a classification suggested for developing countries^33^, which conforms to the recommendations of International League Against Epilepsy (Guidelines for epidemiologic studies on epilepsy. Commission on Epidemiology and Prognosis, International League Against Epilepsy 1993). ‘Active epilepsy’ was defined as epilepsy with one or more seizures within five years of the interview, regardless of antiepileptic drug treatment (International League Against Epilepsy 1993).

### Diagnostic criteria for neurocysticercosis

Neurocysticercosis diagnosis was based on the modified criteria of Del Brutto et al*.*^34^ and the experience on Indian patients^35^. The modifications suggested by Garg et al., were considered because of the high incidence of pulmonary tuberculosis in the tea gardens of Assam^36^; to help differentiate between cysticercus granuloma and tuberculoma. Clinical observations, neuroimaging, immunological and epidemiological data were used to establish cases into either definitive or probable neurocysticercosis.

### CT scan of the brain

A contrast-enhanced CT scan of the brain was performed for the patients with confirmed active epilepsy. The brain parenchymal cysticerci were initially classified into three groups as suggested by Carpio and Escobar^37^ which was further classified into four histopathological stages as suggested by Escobar^38,39^ and Salgado^40^—Active (vascular), transitional (colloidal vascular and granular vascular) and inactive/calcified (nodular calcified). Cystic lesions with a scolex were considered as definite lesions of neurocysticercosis. Cystic lesion without a visible scolex, calcifications in the brain parenchyma and single or multiple rings or nodular enhancing lesions were grouped as lesions highly suggestive of NCC. A diagnosis of hydrocephalus or enhancement of the leptomeninges was considered compatible with neurocysticercosis^41^. Single calcifications were considered compatible only after other forms of granulomatous diseases, mainly tuberculosis were clinically ruled out.

### Enzyme electro-immune transfer blot analysis (EITB)

For EITB assay, we used cystic fluid obtained from cysticerci collected from locally available infected pigs. Immunoblotting was carried out as described earlier^42^ with slight modification. A sample was considered positive if one or more bands (< 50 kDa) were detected.

### Seroprevalence of IgG antibodies against *Taenia solium* metacestode

A total of 987 serum samples were available for the detection of specific IgG antibodies against *Taenia solium* metacestode. All samples were tested using an in-house ELISA, and the absorbance was measured at a wavelength of 492 nm using a microplate reader. The sensitivity, specificity, positive and negative predictive value of IgG ELISA was calculated using sera samples from confirmed neurocysticercosis cases and sera from patients infected with other parasitic diseases. In our internal testing, the IgG ELISA we applied had a 100% sensitivity when used to classify NCC in patients with multiple (Active and mixed) cysticerci in the brain parenchyma (our unpublished data).

### Diagnosis of taeniasis

The diagnosis of taeniasis was made by stool microscopy. From 1028 participants, 497 faecal samples were available. Direct smears were prepared and examined for the presence of *Taenia* eggs. A sample was considered negative if no *Taenia* eggs were visualized in three consecutive smears from the same sample. Individuals with *Taenia* eggs were treated as part of this study under the supervision of a clinician. Although microscopic examination does not distinguish *T. solium* and *T. sagina*ta infections, this limitation was alleviated by the fact that none of the participants reportedly consumed beef.

### Data analysis

Data were compiled and analyzed using the IBM SPSS statistical software, version 16.0 (SPSS Inc., Chicago, IL, USA). The results for continuous variables are represented as median (interquartile range) or mean (standard deviation). The results for categorical variables are presented as numbers. For analyses, study participants were segregated into four age groups (< = 10, 11–19, 20–39, >  = 40 years). A chi-square test was performed to assess proportional differences across select categorical variables (https://www.medcalc.org/calc/comparison_of_proportions.php, last accessed on 25.03.2020). Potential risk factors for neurocysticercosis were assessed using univariate logistic regression analysis and the probable associations thus identified were included in the multivariate logistic regression analysis. For multivariate regression models, crude odds ratios (cOR), adjusted odds ratio (aOR) and 95% confidence intervals (CI) for each independent variable were calculated. All statistical tests were two-tailed. A probability (*p*) value less than 0.05 was used as the level of significance.

### Ethics approval

The Institutional ethical committee of Regional Medical Research Centre, NE. Region (Indian Council of Medical Research) Dibrugarh, approved the study (Certificate Reference Number: RMRC/Dib./Adm-6/2006-07/1893). The study was conducted in accordance with relevant guidelines/regulations. All participants and their parents (or guardians) gave written informed consent before their inclusion in the study.

## Data Availability

The data that supports our findings are available from the corresponding author on reasonable request.
